# Differential Regulation of the Variations Induced by Environmental Richness in Adult Neurogenesis as a Function of Time: A Dual Birthdating Analysis

**DOI:** 10.1371/journal.pone.0012188

**Published:** 2010-08-16

**Authors:** María Llorens-Martín, Gonzalo S. Tejeda, José L. Trejo

**Affiliations:** 1 Centro de Investigación en Red en Enfermedades Neurodegenerativas (CIBERNED), Madrid, Spain; 2 Cajal Institute, Consejo Superior Investigaciones Científicas (CSIC), Madrid, Spain; 3 Centro de Investigación en Red en Enfermedades Neurodegenerativas (CIBERNED), Madrid, Spain; Medical College of Georgia, United States of America

## Abstract

Adult hippocampal neurogenesis (AHN) augments after environmental enrichment (EE) and it has been related to some of the anxiolytic, antidepressant and neuroprotective effects of EE. Indeed, it has been suggested that EE specifically modulates hippocampal neurogenic cell populations over the course of time. Here we have used dual-birthdating to study two subpopulations of newborn neuron in mice (Mus musculus): those born at the beginning and at the end of enrichment. In this way, we demonstrate that while short-term cell survival is upregulated after an initial 1 week period of enrichment in 2 month old female mice, after long-term enrichment (2 months) neither cell proliferation nor the survival of the younger newly born cell populations are distinguishable from that observed in non-enriched control mice. In addition, we show that the survival of older newborn neurons alone (i.e. those born at the beginning of the enrichment) is higher than in controls, due to the significantly lower levels of cell death. Indeed, these parameters are rapidly adjusted to the sudden cessation of the EE conditions. These findings suggest both an early selective, long-lasting effect of EE on the neurons born in the initial stages of enrichment, and a quick response when the environment again becomes impoverished. Therefore, EE induces differential effects on distinct subpopulations of newborn neurons depending on the age of the immature cells and on the duration of the EE itself. The interaction of these two parameters constitutes a new, specific regulation of these neurogenic populations that might account for the long-term enrichment's behavioral effects.

## Introduction

Newborn neurons in the adult dentate gyrus (adult hippocampal neurogenesis, AHN) display specific transient electrophysiological properties while differentiating [1]. When these newborn cells are removed genetically, animals display impaired capacities for learning and memory [2], [3], [4], as well as certain dysfunctions related to mood and depressive disorders [5], [6], [7], [8]. The details of the molecular regulation of AHN are still being studied [1], [9], [10], [11], [12], [13], [14], [15], including the effects of physical exercise and environmental enrichment [13], [16], [17], [18].

Environmental enrichment (EE) influences certain behaviors [16], [17], [19] and it modulates AHN [20], [21], [22], [23], [24], [25], [26], [27], [28], [29] distinctly over time. It has been shown that EE produces initially a large increase in survival and proliferation of cells during the first 24–72 hours after the cells are birth labeled [27], [30], [31]. Subsequently, there is a period (4-days to 3-week old cells) when survival has been reported comparable to that of control animals by a number of works [17], [24], [28], [29], [30], while other work has shown that the relevant aspect is not only the age of the new cell to define the critical time window, but whether the enrichment has just begun or has been present for a longer time [32]; finally, there is a number of works reporting that long-term EE greatly augments cell survival (≥3-week old cells: [24], [28], [29], [33], [34]. All these lines of evidence point to the hypothesis that the effects of physical-cognitive activity on neurogenesis depend on the interaction of two critical parameters: the age/differentiation status of the immature neuron plus the time the individual is under the effects of an enriched environment. To test this hypothesis, we have labeled two completely separated subpopulations of cells into every animal, in order to see the distinct effect of the same EA stimuli on two spatially contiguous, temporally separated neuron subpopulations (dual birthdating). We have used this approach both for the short- and long-term EA, and also after the cessation of a long-term EA.

How EE affects the generation and survival of neurons is relevant to the development of pharmacomimetics of physical activity [30], [31], [33], [35], [36], [37], [38], [39], [40], [41], [42], [43], [44]. This is particularly relevant if we consider that certain therapies have different neuroprotective effects when administered on naïve or older individuals [25], [39] or, more importantly, when individuals have previously been subjected to stressful events [6]. In this way, studying the details of EE effects over time should provide us with information about the possible windows of sensitivity to the positive effects of EE.

## Materials and Methods

### Animals

In this study, we used 60 adult C57/BL6J female mice (8 weeks of age, Harlan Laboratories) that were housed at 22±1°C on a 12/12 h light/dark cycle, with *ad libitum* access to food and water. Mice were kept under standard laboratory conditions in accordance with European Community Guidelines (directive 86/609/EEC). All animals were handled in strict accordance with good animal practice as defined by the national animal welfare bodies (Cajal Institute and CSIC (Consejo Superior de Investigaciones Científicas), the High Council of Scientific Investigation), and all animal work was approved by the appropriate committee (the Bioethics Committee of our institution, the Cajal Institute and CSIC, -the high council of scientific investigation-, through the approval certificate number BFU2007-60195 issued on June 7, 2007).

### Experimental design

**Figure 1:**
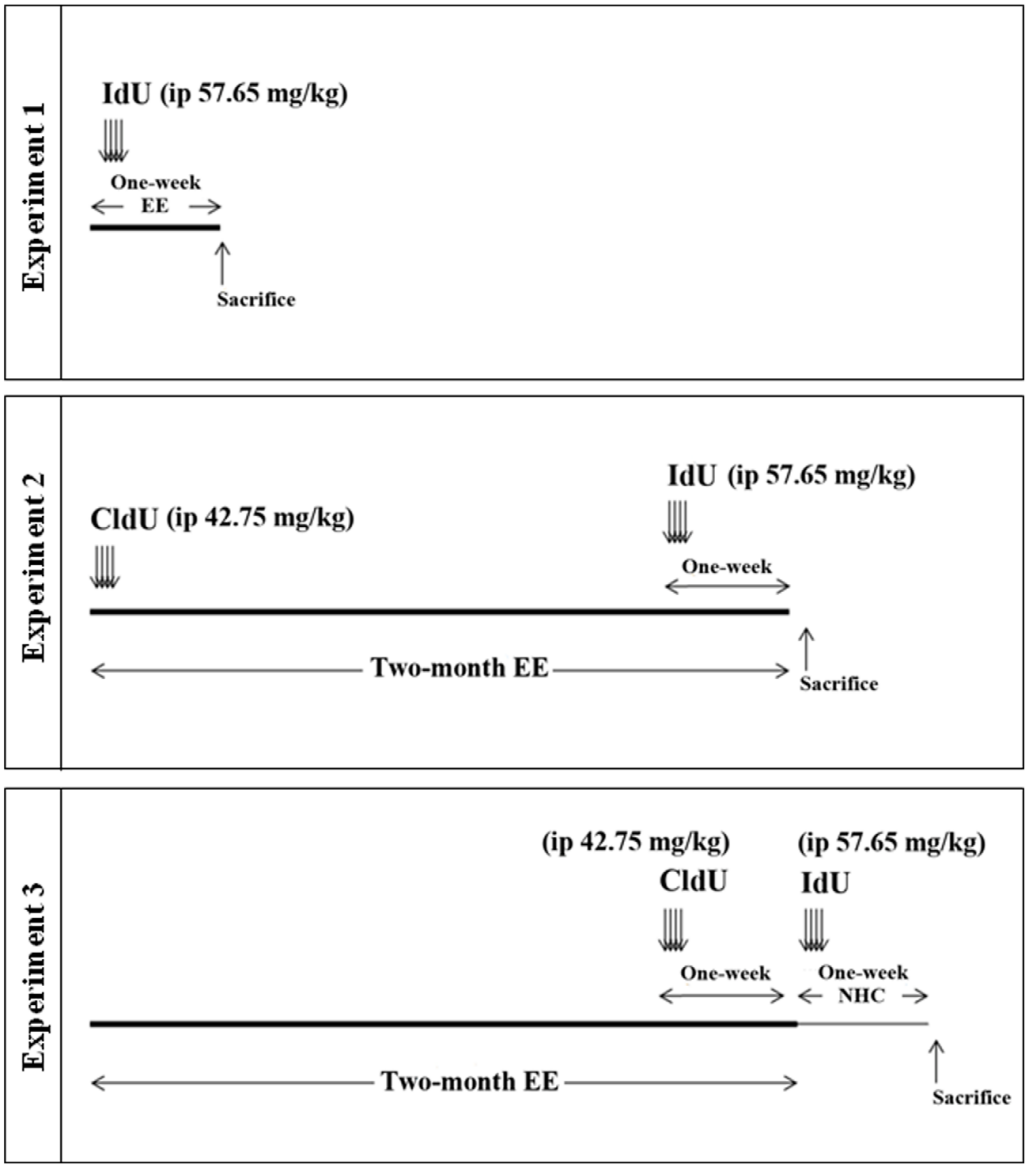
Timeline and design of experiments. CldU and IdU were injected intraperitoneally into the same animals in Experiments 2 and 3. EE, environmental enrichment. NHC, normal housing conditions.

After one week in quarantine the mice were randomly distributed into three experiments, as follows (see figure 1): Experiment 1; 10 mice (8 weeks old) were exposed to a one-week Environmental Enrichment (EE) protocol, in order to be compared with 10 control mice (dedicated) housed in standard laboratory conditions. Experiment 2; 10 mice were exposed to a two-month EE protocol, and they were compared with 10 control mice housed in standard laboratory conditions (dedicated). Experiment 3; 10 mice were exposed to the same two-month EE protocol, and then were returned to standard housing conditions during one more week. These mice were compared with 10 control mice housed in a nine-week period of standard laboratory conditions.

### CldU and IdU injection protocol

Mice belonging to Experiment 1 received one injection of 5-Iodo-2'-deoxy-Uridine (IdU: i.p. 57.65 mg/Kg bw) per day during the first four days of the EE. Experiment 2 mice received one injection per day of 5-Chloro-2'-deoxy-Uridine (CldU (i.p. 42.75 mg/Kg bw)) during the first four days of the EE protocol, and one injection per day of IdU (i.p. 57.65 mg/Kg bw) during the first four days of the last week of the enrichment (the last week of the experiment). Mice belonging to the Experiment 3 received one injection per day of CIdU (i.p. 42.75 mg/Kg bw) during four days at the beginning of the last week of the enrichment, and one injection per day of IdU (i.p. 57.65 mg/Kg bw) during four days just after finishing the enrichment (one week before they were sacrificed). The doses of the thymidine analogues administered and the survival times are summarized in Table 1.

**Table 1:** Dosages of the thymidine analogues used in each experiment, indicating both the dose injected to the animals and the survival time after the last injection to the moment of sacrifice.

Experiment	Thymidine analogue	Dose (i.p. mg/Kg)	Survival period
Experiment One	IdU	57.65	4–7 days
Experiment Two	CldU	42.75	2 months
Experiment Two	IdU	57.65	4–7 days
Experiment Three	CldU	42.75	11–14 days
Experiment Three	IdU	57.65	4–7 days

Equimolar doses to 50 mg/kg BrdU were calculated.

The doses of the thymidine analogues used were based on the equimolar doses of BrdU administration and accordingly, we calculated CldU and IdU equimolecular doses to 50 mg/kg BrdU. To determine whether such doses were appropriate to achieve the quality of labeling required in the experiments, we previously carried out a series of experiments in which both thymidine analogues were injected into the same animal and the cells were labeled after different survival times in different groups of animals. We injected CldU and IdU at different times in the same animals and then sacrificed them after 24 h and 2 weeks. When these equimolar doses were used, only slightly fewer cells were labeled than when using BrdU, although these numbers cannot be directly compared with that of BrdU administration (according to recent findings [2] [45]. In terms of the quality of labeling, the cells were consistently labeled in decreasing numbers as the survival time increased with both analogues, as would be expected. Moreover, the number of CldU^+^/IdU^+^ double-labeled cells in the same animal also decreased as the time between the injections of each analogue augmented. We also varied the order of the injections with some animals injected first with CldU and then with IdU, and other animals in the opposite order. The mentioned quality of the labeling was not affected whatever the order of injection. The cell numbers we obtained after 24 h survival when either CldU or IdU was injected, and the cell numbers obtained after 2 weeks survival after injecting either CldU or IdU, were consistently similar.

### Environmental enrichment

We used an EE protocol [46] involving classical toys and objects, and a running wheel [47]. This paradigm has proved useful to modulate some aspects of AHN [17]. Control and enriched mice were housed differently in these experiments. Thus, non-enriched mice were housed in groups of five in transparent Plexiglas cages (20×22×20 cm) under standard laboratory conditions. Mice subjected to EE were housed in groups of 10 (animals) in large transparent Polycarbonate cages (55×33×20 cm, Plexx Ref. 13005) that were divided into floors connected by a stairway. All enriched cages were equipped with different types of running wheels. Every two or three days all the toys, bedding material and the running wheel were replaced with different ones. The mice constantly had at their disposal toys of different shapes, sizes, materials and surface texture. The bedding material used ranged from newspapers, sheets of paper, sawdust and cloth, to other non-toxic materials. The toys used included plastic non-edible ones, hard plastic toys, small plastic animals, gumabones (Plexx Ref. 13110), tips, pet balls, crawl balls (Plexx Ref. 13121), a transparent polycarbonate mouse igloo (Plexx Ref. 13100), polycarbonate rodent tunnels (Plexx Ref. 13102) and others. Every other day, a set of 10–20 different toys and new bedding material was introduced into the cages and in general, the complexity of the cage environment was changed completely. Spirals, boxes, tubes, tunnels and hiding places guaranteed that each time the materials were replaced the resulting environment had a completely new-look. In this manner, the mice were continuously exposed to a totally new and unexpected environment during all the experiment.

### Sacrifice and histology

Twenty four hours after the last day of the EE protocol, the mice were completely anaesthetized with an intraperitoneal penthobarbital injection (EutaLender, 60 mg/kg bw) and they were then transcardially perfused with saline. Their brains were removed and post fixed overnight in 4% paraformaldehyde in phosphate buffer (PB).

Coronal sections of the brain were obtained on a Leica VT1000S vibratome (50 µm thick sections). These serial vibratome sections of the hippocampal formation were collected individually in 96-multiwell culture plates. Series of the brain slices were made up randomly of one section from every 9^th^ for the immunohistochemical analysis. A randomly chosen series was used for Nissl Staining to calculate the total volume of the dentate gyrus in each animal.

The slices were initially pre-incubated in PB with 0.5% Triton X-100 and 0.1% bovine serum albumin (BSA), and then dual immunohistochemistry was performed as described previously [48]. The primary antibodies used here (at the dilutions indicated in brackets) were the following: rat anti-CldU antibody (Accuratechemicals 1∶500), mouse anti-IdU antibody (BD Biosciences 1∶500), rabbit anti-phospho-histone 3 (pH 3) antibody (Upstate 1∶500), goat anti-doublecortin antibody (Santa Cruz 1∶500), rabbit anti-calretinin antibody (Swant 1∶3,000); guinea Pig anti-vesicular glutamate transporter1 (vGlut1) (Chemicon 1∶2,500), mouse anti-glutamic acid decarboxylase (GAD) (Developmental Hybridome Bank 1∶500), rabbit anti-cfos antibody (Calbiochem 1∶10,000), rabbit anti-fractin antibody (BD Biosciences 1∶500; fractin is an actin fragment cleaved by caspase). Secondary Alexa-conjugated antibodies from Molecular Probes were also used at a final concentration of 1∶1,000 to detect the primary antibodies as follows: Alexa 594 conjugated donkey anti rabbit for the anti-pH3 antibody, Alexa 568 conjugated donkey anti rabbit for the anti-cfos antibody, Alexa 488 conjugated donkey anti rat for the anti-CldU antibody, Alexa 594 conjugated donkey anti mouse for the anti-IdU and anti GAD antibodies, Alexa 594 conjugated donkey anti goat for the anti-doublecortin antibody, Alexa 488 conjugated donkey anti rabbit for the anti-calretinin antibody, biotin-conjugated goat anti Guinea pig for the anti-vGlut1 antibody followed by incubation with a Alexa 488 conjugated Strepatividin. All sections were counterstained with DAPI (Calbiochem, 1∶1,000). The incubation period ranged from 24–48 hours at 4°C for primary antibodies, 24 hours at 4°C for secondary antibodies and 10 minutes for DAPI incubation.

### Cell Counting

The total numbers of pH 3, c-fos, CldU and IdU positive cells were counted under an optical fluorescence microscope (Leica DMI 6000 B, oil immersion 40× objective) using the optical disector method. Briefly, a standard optical disector protocol [49] was used to measure the cell density of the labeled cells in the dentate granule cell layer. An unbiased sampling method according to the principles of Howard & Reed [49] was used to select the positions where the disector was applied in every section. Next, a mean cell density for every animal was calculated. Independently, the volume of the granule cell layer of every animal was measured by means of the Cavalieri method (point grid) by using Nissl sections. Finally, the total cell number for every population was calculated by multiplying the mean cell density by the granule cell layer volume animal by animal. Group means of total cell number were obtained to compare statistically the data. The total numbers of immature doublecortin and calretinin positive cells and of mature granule neurons were analyzed by applying a physical disector method developed for confocal microscopy (Leica TCS SP5, oil immersion 63× objective) as described previously [48]. We have applied a physical disector method [50] introducing some variations according to the basic principles of Howard and Reed [49] and using the “unbiased brick” principle of the 3D disector, as previously published [17], [41], [48], [51]. We counted the cells in each pair of confocal sections using the first as a reference section and the other as a “look-up” section, and then their identities were swapped. The cells were marked on a grid superimposed on the computer screen and they were then identified in successive images of the disector to assure that each single cell was no longer counted. We implemented the traditional optimization of the disector by registering successive pairs of sections in the “vertical” (z) axis with the aid of a confocal microscope. The cell counts and the size of the dissector used to estimate the density, were given by the general formulae N = ∑C/Vf, where N = estimate of numerical density, ∑C = sum of cells counted, and Vf =  volume of the disector. For the immature granule neurons, the disector was implemented as the number of cells per unit area of the SGL (see below), substituting Vf for Af =  area of the dissector. The average distance between the confocal planes throughout the study was in the range of 1.7–2 µm, as 6–9 confocal planes were recorded for all the points measured. To ensure uniform random sampling, we considered that the supra- and infrapyramidal blades of the DG in all the sections within a series generated a continuous “line,” throughout the rostro-caudal extent of the entire DG. This “line” was then measured with a digital tablet and assigned an arbitrary unit length. A random number table was then used to generate the exact points to obtain the stacks. This means that every single point of the GCL has the same chance of being selected for the confocal stack of images. For the total number of granule cells, the physical dissector was applied to sections stained with propidium iodide so that all nuclei in the GCL were counted (excluding those nuclei that, upon observation, resembled erythrocytes). The cell density was then multiplied by the total volume of the GCL estimated by the Cavalieri method (point grid).

A different method was used for the cells expressing immature markers (doublecortin and/or calretinin). At each point of the section to be registered, we counted all the immature cells in the reference area. The reference area is a square with one side that lies on the “line” of the SGZ. By applying the simple rule of dividing the number of immature cells counted by the length of the “subgranular” line, we obtained a reliable estimate of the cell density by “unit of SGZ.” The total number of immature cells was obtained by simply multiplying the cell density by the total extension of the SGZ. This total SGZ extension was measured using a semiautomatic system (ImageJ v.1.33, NIH, USA, http://rsb.info.nih.gov/ij/) with the series of images from DAPI-stained sections. We then drew the SGZ below the internal side of the GCL on the computer screen and measured the length of the resulting lines.

The total area occupied by either glutamatergic or gabaergic terminals in the inner molecular layer was obtained as described previously [52] and measured with the help of a semiautomatic image analysis software (ImageJ, v. 1.33, NIH, Bethesda, MD, USA, http://rsb.info.nih.gov/ij). This same software was used to count the total number of immature and mature granule neurons.

### Statistical Analysis

Cell counts were analyzed by applying one-way ANOVA to compare between the control and enriched animals from each experiment. The correlation between different cell subpopulations was analyzed using the bivariate correlations test. Statgraphics Plus 5.1 (Statistical Graphics 1994) software was used to analyze the cell numbers and SPSS 17.0.1 software (SPSS, 1989; Apache Software Foundation) was used to analyze the correlations between the different populations.

## Results

In order to determine how EE conditions regulate cell survival, proliferation and death over time, as well as the influence of terminating a period of EE, we studied these parameters in female mice, analyzing the cells born in the adult in the GCL of the hippocampal dentate gyrus under three different experimental paradigms.

**Figure 2:**
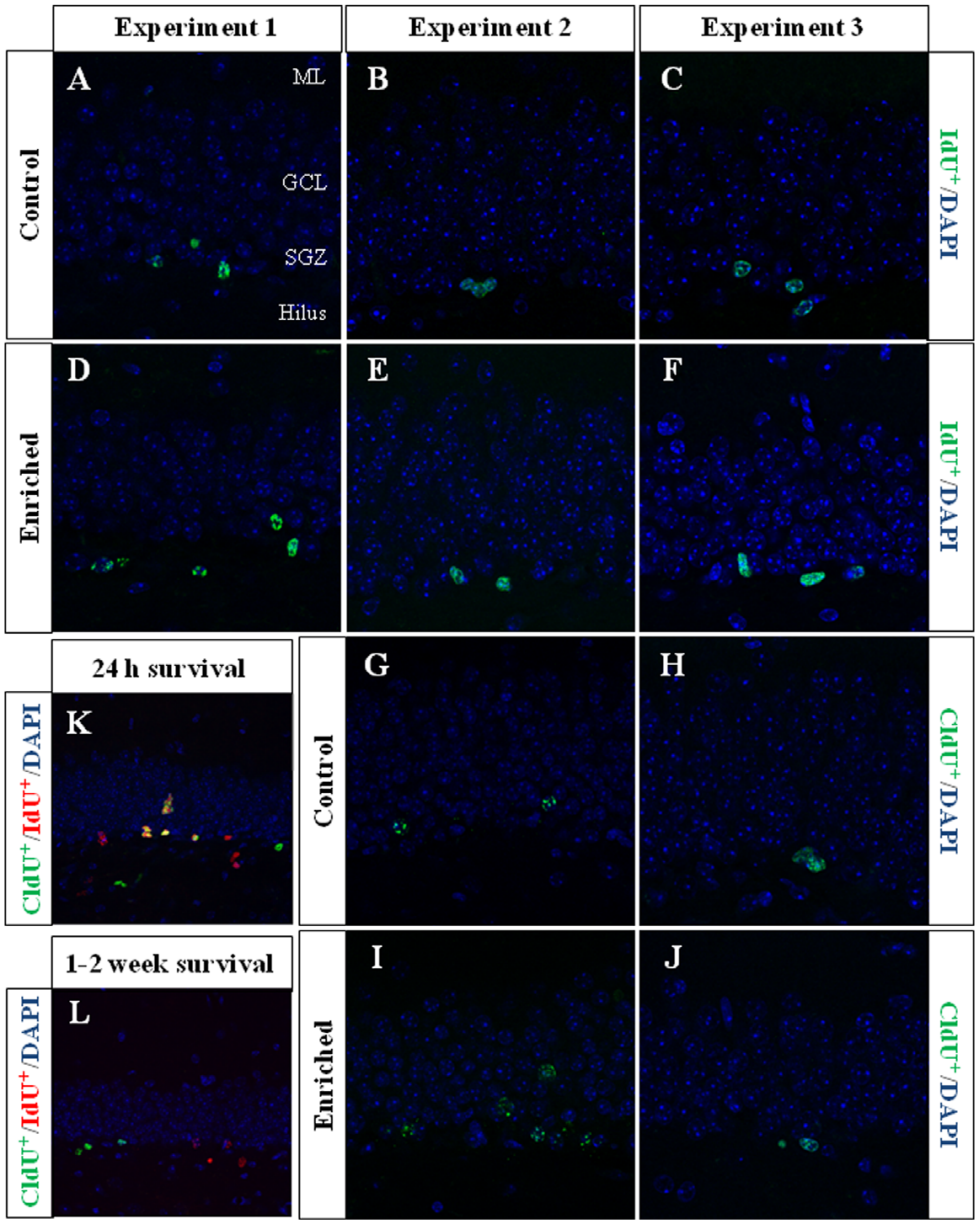
Representative images of CldU^+^ and IdU^+^ cells. **A–J**, Representative images of the cells labeled in the different experiments. **K–L**, Representative images of CldU and IdU labeling of the animals used in the preliminary experiment, in which BrdU-equimolar dosages of CldU and IdU were injected to animals of the same strain, sex and age used for the current experiments. **A–F**, Immunohistochemistry for IdU showing higher IdU^+^ cell numbers in enriched animals (**D**) compared with control normal-housed animals (**A**) only in Experiment 1. **G–J**, Immunohistochemistry for CldU (older cells compared to the younger IdU cells). We found an increased CldU^+^ cell number in enriched animals (**I**) compared with controls (**G**) in Experiment 2. As the injection of different thymidine analogues separated by more than one day led to no overlapping of the labeling as seen on the representative images (**L**), in the present work a set of different sections to those ones used for IdU immunohistochemistry were used to label CldU^+^ cells in both Experiments 2 and 3. ML, Molecular layer. GCL, Granule cell layer. SGZ, Subgranular zone.

**Figure 3:**
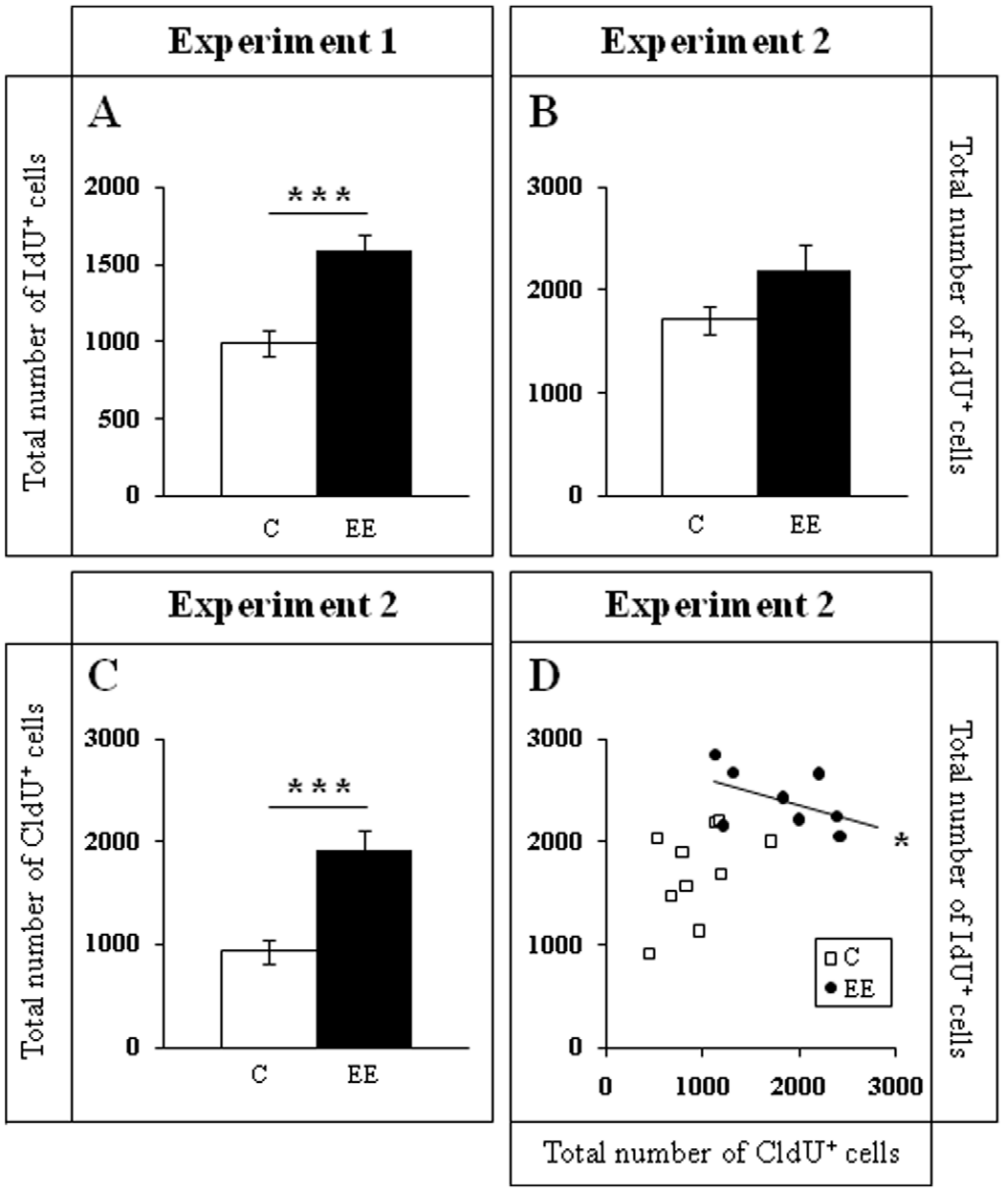
Data from cell counts corresponding to CldU/IdU labeling. **A–B**. IdU cell number. The number of 4-to-7 day old IdU^+^ cells in Experiment 1 is significantly higher in enriched animals than in control mice after only 1 week of enrichment (A). After 2 months of EE (Experiment 2) the increase in the survival of 4-to-7 day old IdU^+^ cells is not maintained (**B**). The number of 2-month old CldU^+^ cells labeled in the same animals as IdU^+^ cells in Experiment 2, is higher in enriched than in control animals after 2 months of EE (**C**). In fact, a significant negative correlation was found between CldU^+^ and IdU^+^ cells only in the enriched animals from Experiment 2. The higher the number of CldU^+^ cells (older), the lower the number of IdU^+^ cells (younger) labeled in the same animals (**D**). *** p≤0.001; * 0.01≤p≤0.05. C Control; EE Environmental Enrichment. For D, □ control mouse; • enriched mouse.

**Figure 4:**
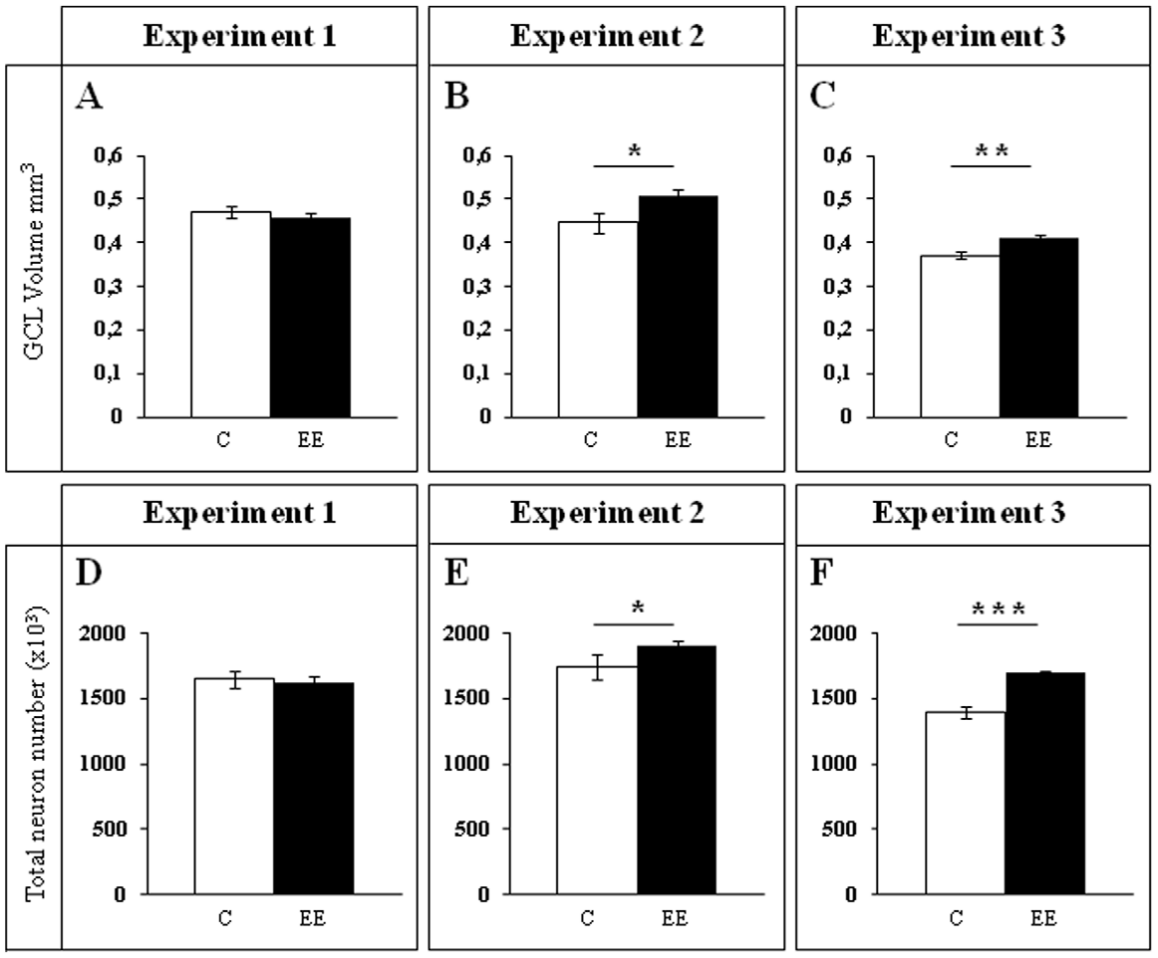
Estimates of GCL volume and total cell number in the GCL. **A–C**, GCL volume estimation (mm^3^). **D–F**, Total cell number in the GCL stained with DAPI (10^3^ cells). Two month EE significantly increased both the volume of the GCL (**B**,**C**) and the number of mature granule neurons (**E**,**F**), when compared to control animals (Experiments 2 and 3). One week of EE was not able to increase these parameters (**A**,**D** Experiment 1). *** p≤0.001; ** 0.001≤p≤0.01; * 0.01≤p≤0.05. C Control; EE Environmental Enrichment.

**Figure 5:**
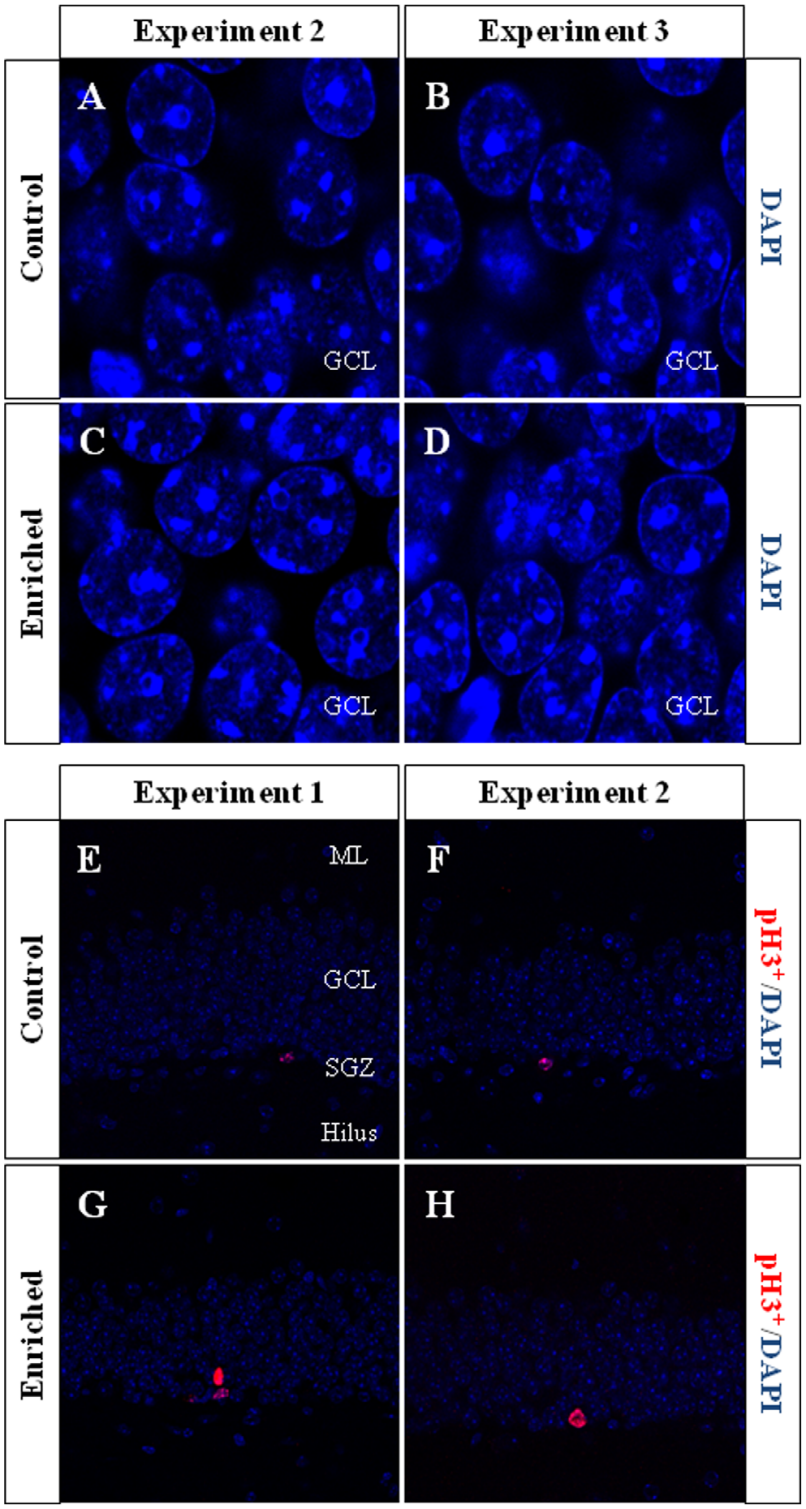
Representative images of mature granule neurons and phospho-histone (pH3)^+^ cells. **A–D**, DAPI stained cells in the GCL. We found a higher number of clearly identifiable DAPI stained mature granule neurons (≈97% of the total cell number in the GCL, clearly distinguishable from blood cells or other cell types) in the GCL of enriched animals (**C**,**D**), when compared with controls (**A**,**B**) only after 2 months of EE (Experiments 2 and 3). **E–H**, pH 3 immunohistochemistry. We found a significantly higher number of pH 3^+^ cells in enriched animals of Experiment 1 (**G**) than in control animals (**E**). All pH 3^+^ cells were situated in the SGZ. ML Molecular layer; GCL Granule cell layer; SGZ Subgranular zone.

**Figure 6:**
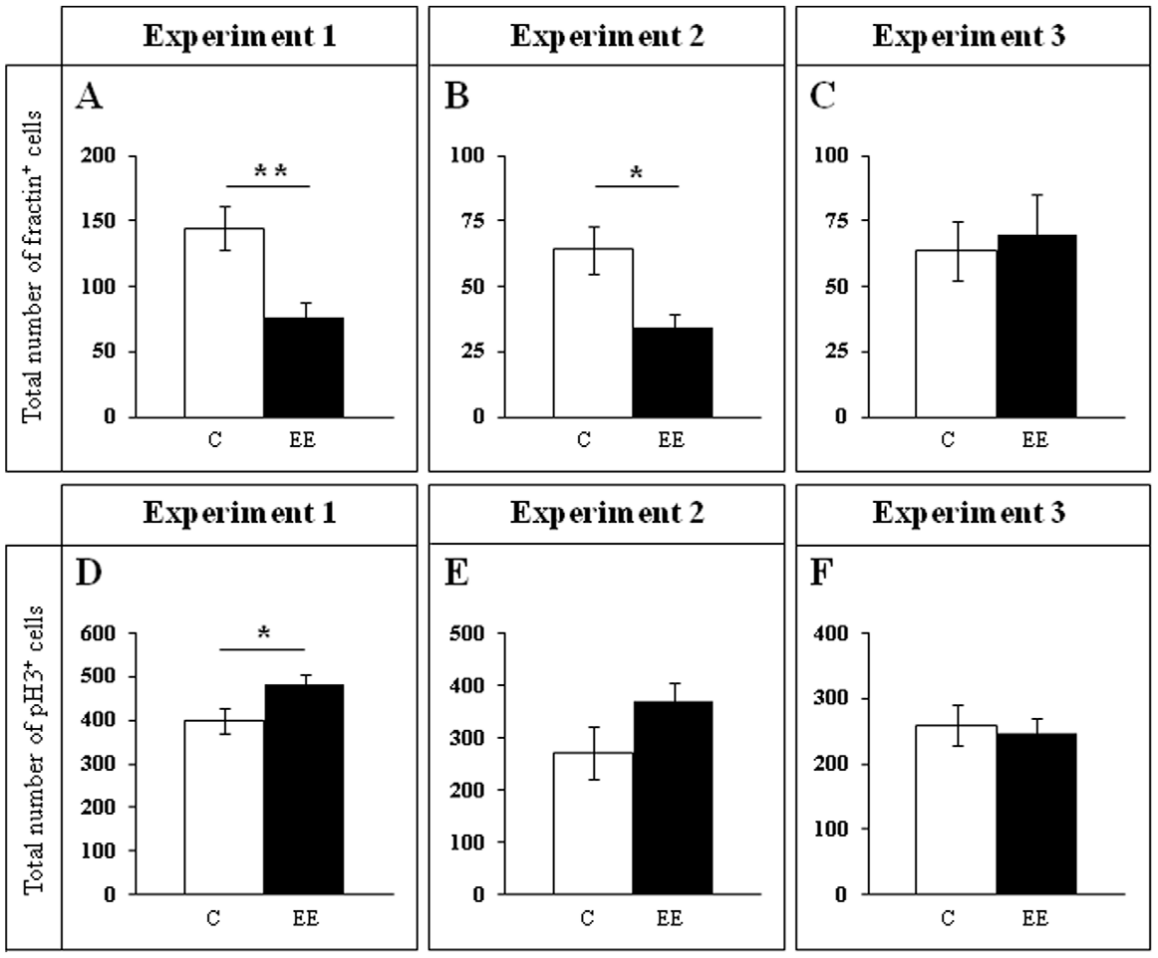
pH 3 and fractin cell counts. **A–C**, Fractin^+^ cell number. During the EE period, a lower number of fractin^+^ cells was found in enriched animals when compared to controls (**A**,**B**; Experiments 1 and 2). Only 1 additional week in normal housing conditions was sufficient to normalize the size of this cell subpopulation (**C**; Experiment 3). **D–F**, pH 3^+^ cell counts. Only at the beginning of the EE period (one week, Experiment 1) the number of pH 3^+^ cells is higher in enriched than in control animals (**D**). ** 0.001≤p≤0.01; * 0.01≤p≤0.05. C Control; EE Environmental Enrichment.

**Figure 7:**
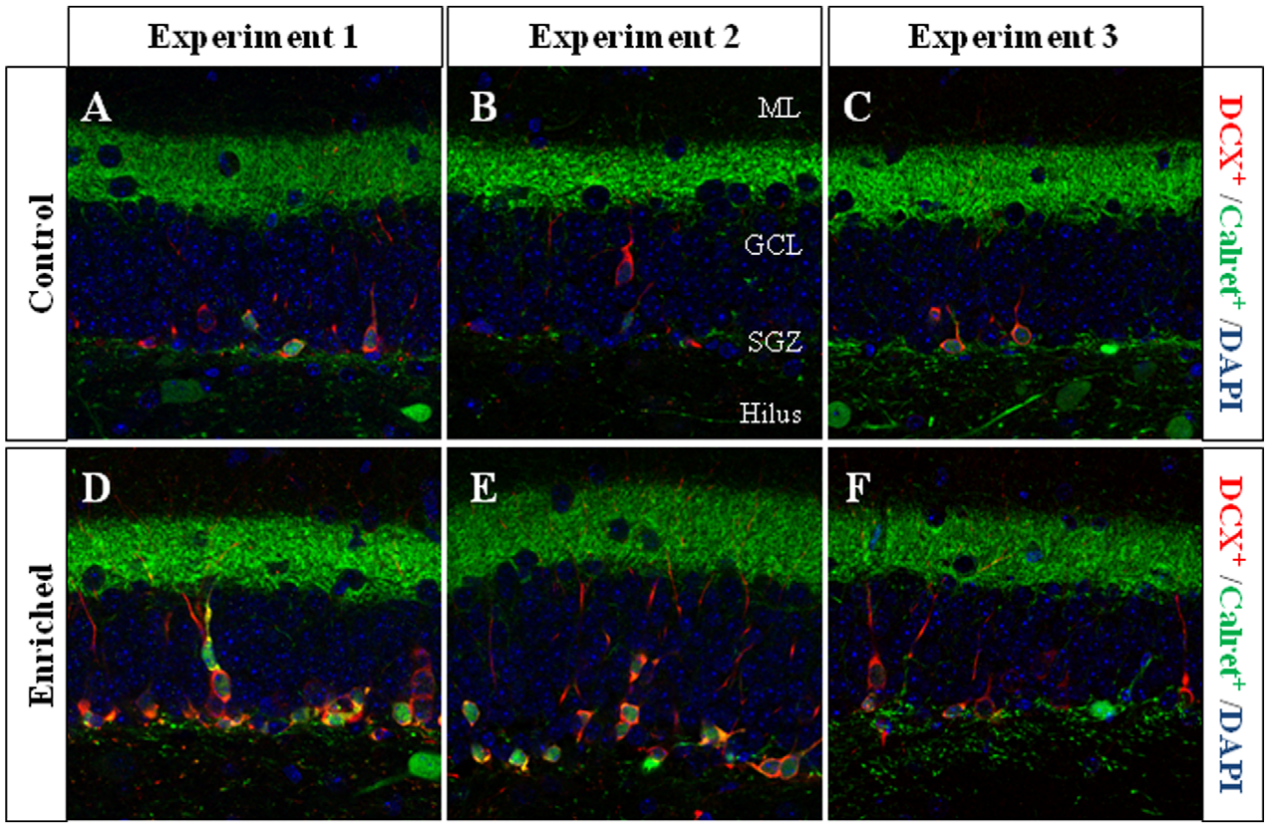
Representative images of doublecortin (DCX) and calretinin (Calret) immunohistochemistry. During the EE intervention, enriched animals have shown a higher number of DCX^+^ cells when compared to control animals. In these images, DCX^+^/Calret^−−−^, DCX^+^/Calret^+^, and DCX^−−−^/Calret^+^ cells can be detected. Only the cells in the SGZ or GCL were counted. ML Molecular layer; GCL Granule cell layer; SGZ subgranular zone.

**Figure 8:**
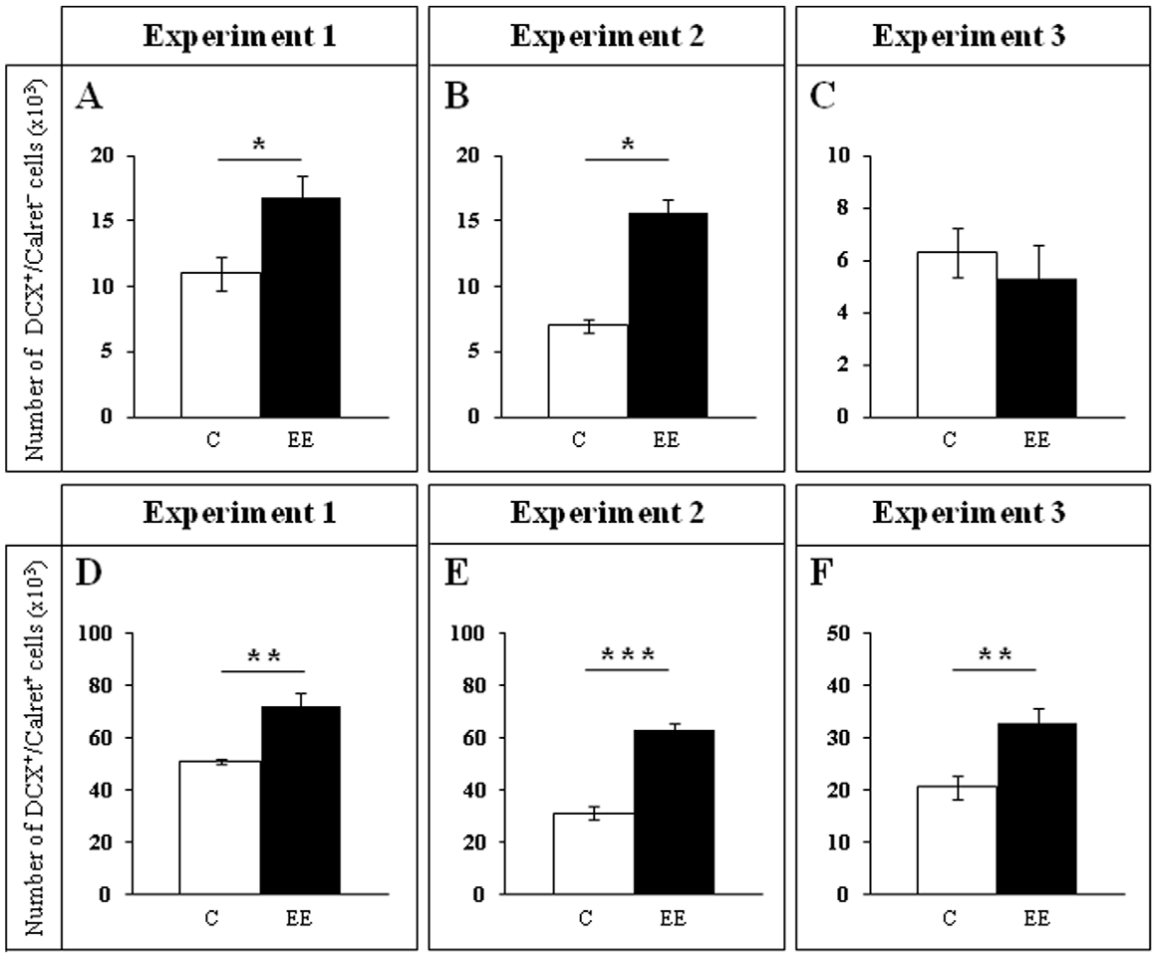
DCX/Calret cell number. A–C, DCX^+^/Calret^−−−^ cell counts. The smaller and younger subpopulation of DCX^+^ cells was significantly higher in enriched animals when compared to controls during the EE intervention (**A**, **B**; Experiments 1 and 2). After one week in normal housing conditions and the termination of EE (Experiment 3), the numbers were no longer significantly different (**C**). **D–F**, DCX^+^/Calret^+^ cell counts. The larger and more differentiated subpopulation of DCX^+^ cells was significantly higher in the three experiments. *** p≤0.001; ** 0.001≤p≤0.01; * 0.01≤p≤0.05. C Control; EE Environmental Enrichment.

**Figure 9:**
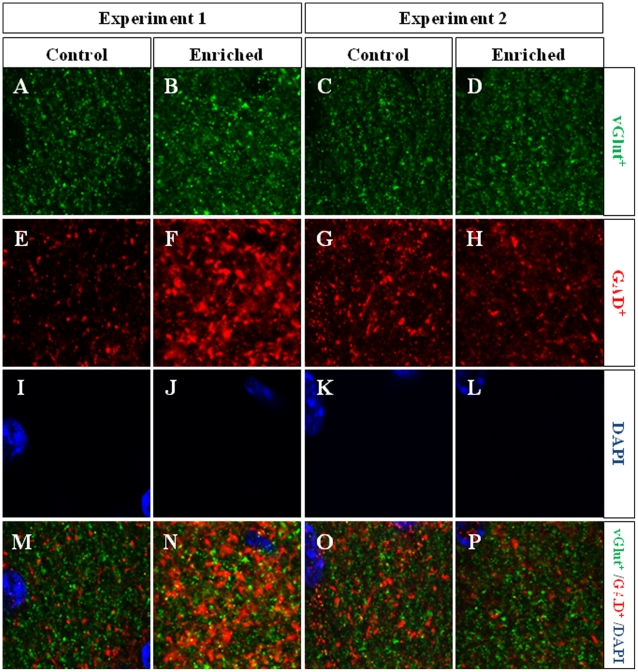
Representative images of vGlut and GAD immunohistochemistry. In all cases, the inner molecular layer of the dentate gyrus was measured, and images were recorded separately for each confocal channel, i.e. vGlut^+^ boutons in green (**A–D**), GAD^+^ boutons in red (**E–H**). The external row of granule neurons in the GCL can be seen labeled with DAPI (**I–L**). There is no co-localization of excitatory and inhibitory boutons at any site. The GAD^+^ area is more extensive in enriched mice in Experiment 1 (**F**) than in controls (**E**). Merged images can be seen at the bottom of the figure (**M–P**). The same magnification has been used for all images.

**Figure 10:**
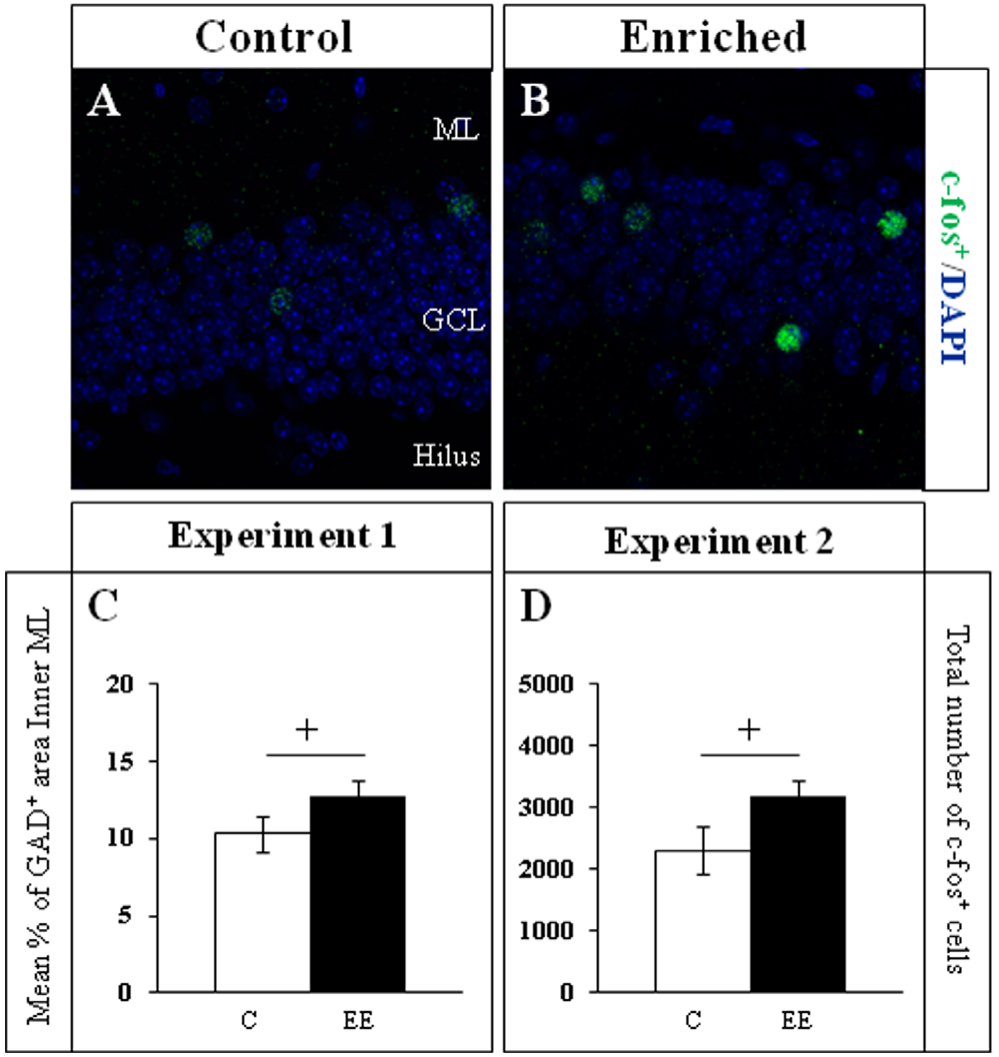
Cellular c-fos expression in the GCL, and GAD immunohistochemistry for synaptic boutons in the inner molecular layer. **A**,**B**,**D**. c-fos immunohistochemistry. A clear tendency towards more c-fos^+^ nuclei can be seen in the GCL of enriched animals (**B**) when compared to controls in Experiment 2 (**A**; cell counts in **D**). **C**. The GAD^+^ area was measured in confocal images similar to those depicted in Figure 9. A strong tendency towards a higher mean percentage of GAD^+^ area was found in enriched animals when compared to the controls only in Experiment 1. + 0.05≤p≤0.075. C Control; EE Environmental Enrichment; ML Molecular layer; GCL Granule cell layer.

In Experiment 1 we analyzed the cell proliferation and survival of new 4-to-7 day old neurons born at the beginning of a one-week period of EE. We found that EE significantly increased the total number of IdU^+^ cells in the GCL (≈60%, F_(1,16)_ 20.422, p<0.0001; figures 2 and 3). As expected, a one-week period of EE was unable to significantly increase either the GCL volume (F_(1,17)_ 1,850, p = 0.192; figure 4), or the total number of mature granule neurons (F_(1,18)_ 0.166, p = 0.689; figure 4). EE caused a significant increase (≈25%) in the total number of pH 3^+^ cells in the GCL (F_(1,17)_ 4.842, p = 0.0432; figures 5 and 6), and a significant reduction (43%) in the total number of fractin^+^ cells (fractin is an actin fragment cleaved by caspase labeling dying cells; F_(1,18)_ 10.98, p = 0.004; figure 6). The number of immature DCX^+^ cells also increased (figures 7 and 8). The number of DCX^+^/Calret^−−−^ cells in EE mice was ≈60% higher than in controls (F_(1,18)_ 7.438, p = 0.014), while there were ≈40% more DCX^+^/Calret^+^ cells (F_(1,14)_ 9.853, p = 0.007). Interestingly, the area covered by GAD^+^ boutons in the inner-third of the molecular layer had a tendency to increase in EE animals when compared to controls (figures 9 and 10). More importantly, a significant positive correlation (Pearson's P_(1,20,19)_ 0.508, p = 0.026) was found between the area of GAD^+^ boutons and the number of DCX^+^ cells, while no other significant correlations were found between the parameters analyzed here.

In Experiment 2, we analyzed the survival of 4-to-7 day old new neurons born at the end of a two-month period of EE by means of IdU injections and, in the same animals, the survival of 2-month-old neurons born at the beginning of EE and identified by CldU injections (figures 2 and 3). We found that the number of CldU^+^/2-month old neurons increased significantly (100%) when compared to controls (F_(1,17)_ 18.841, p<0.0001), while the number of IdU^+^/4-to-7 day old was similar between EE and control mice (F_(1,17)_ 1.068, p = 0.316). No correlation was found between the area of GAD^+^ boutons and the number of any immature neurons' subpopulation. The GCL volume of EE mice showed a significant increase (≈11% increase; F_(1,17)_ 4.789, p = 0.043; figure 5) when compared to controls, and the total number of granule neurons in the GCL was significantly higher in the EE mice (F_(1,14)_ 8.357, p = 0.015; figures 4 and 5). A particularly relevant finding from this experiment was the significant negative correlation between the number of CldU^+^ and IdU^+^ neurons in the EE mice, whereby the higher the number of CldU^+^ cells, the lower the number of IdU^+^ cells we observed (Pearson's P_(1,19,18)_ 0.534, p = 0.022; figure 3D). Importantly, while cell proliferation in the GCL measured as the number of pH 3^+^ cells was similar between EE and control mice (F_(1,17)_ 2.357, p = 0.143; figures 4 and 6), cell death in the GCL (fractin^+^ cells) showed a significant reduction (≈45%) in EE mice (F_(1,15)_ 7,254, p = 0.017; figure 6). As far as immature granule neurons in the GCL were concerned (figures 7 and 8), we found a significant increase (≈100%; (F_(1,15)_ 114.027, p<0.0001) in the total number of DCX^+^ cells in EE mice with regards to the controls. Specifically, there were a ≈115% higher number of DCX^+^/Calret^−−−^ (F_(1,14)_ 44.487, p<0.0001), and a 100% higher number of DCX^+^/Calret^+^ cells (F_(1,15)_ 94.488, p<0.0001). Interestingly, there was a clear tendency to find more c-fos^+^ cells in the GCL of 2-month EE mice when compared with controls (F_(1,15)_ 3.666, p = 0.075; figure 10), which was not evident after a 1-week period of EE (F_(1,16)_ 1.070, p = 0.316).

In Experiment 3, we analyzed the survival of 4-to-7 day old neurons born immediately after the termination of a two-month period of EE identified by means of IdU injections (i.e. during the first week in the absence of EE). In the same animals, the survival of 11-to-14 day old neurons born during the last week of the 2 month period of EE identified by means of CldU injections (i.e. during the last week under the effects of EE and the first week in the absence of EE; figure 2) was also assessed. As expected taking into account our results from Experiment 2, we found that both CldU^+^/11-to-14 day old neuron number (F_(1,16)_ 0.027, p = 0.870) and IdU^+^/4-to-7 day old neuron number are similar between control and EE mice (F_(1,18)_ 0.086, p = 0.772) after only 1 week in the absence of EE. The relevant finding in this experiment is that while GCL volume (≈10%; F_(1,18)_ 11.174, p = 0.004; figure 5), the total number of mature granule neurons (≈22%; F_(1,16)_ 33.558, p<0.0001; figures 4 and 5) and the total number of DCX^+^ cells (≈35%; F_(1,18)_ 6.364, p = 0.021; figures 7 and 8) consisting mainly of DCX^+^/Calret^+^ cells (≥60%; F_(1,18)_ 11.841, p = 0.003), remained higher in EE mice than in controls after a 2-month EE plus 1 week after termination of EE, neither the number of DCX^+^/Calret^−−−^ (F_(1,17)_ 1.893, p = 0.187) nor the number of fractin^+^ cells in the GCL were dissimilar between the EE and control mice (F_(1,18)_ 0.128, p = 0.725; figure 6). Indeed, there was no significant difference in the number of pH 3^+^ cells either (F_(1,18)_ 0.110, p = 0.744; figure 6).

## Discussion

We have examined whether the duration of EE interacts with the age of the immature hippocampal neurons to define the effect of EE on neurogenesis. We first analyzed the proliferation and survival of 4-to-7 day old new neurons born at the beginning of a one-week period of EE (when the number of older neurons was expected to be normal in previously non-enriched mice). We found that EE significantly increases the number of IdU^+^ cells in the GCL. However, when we analyzed the cell survival of the same kind of cells, but this time born at the end of a 2-month EE (when the number of neurons older than these ones would be expected to be higher after a long-term EE), we found that there were no longer more IdU^+^ cells than in controls. Consistently, we found that the number of older newborn neurons after this period of EE, (2-month-old new neurons born at the beginning of the EE), is significantly higher than in the controls.

It was previously reported that EE induces an early increase in cell survival in 24-to-72 hour old newborn cells [27], [30], [31]. It has also been reported that EE has no effect on the survival of 4 day to 3 week old cells [17], [24], [28], [29], [30], although Tashiro et al. have shown that the relevant aspect is not only the age of the new cell to define the critical time window, but whether the enrichment has just begun or has been present for a longer time [32]; and EE does ultimately induce a long-lasting increased survival of cells older than 3-weeks old [24], [28], [29], [33], [34]. To date, there is no information about the survival of ≈1 week old neurons at the beginning of an EE. Our data point to an early effect of EE on young newborn neural subpopulations. In addition, the survival of those cells under the early effects of EE was better for up to 2 months later. By contrast, this effect was no longer evident on new generations of new neurons born in EE after a long period of time.

Both groups of data are closely related, as a significant negative correlation was found between older and younger cells born under the effects of EE: the higher the number of older newborn neurons, the lower the number of younger ones. As a consequence, it can be considered that by increasing the number of the older surviving cells, EE might have a direct effect on the younger newborn cells, although further studies of these phenomena are still needed. It is interesting to determine whether the influence of EE on immature neurons depends on the time the animals are under the effects of EE. Indeed, it has been shown that the response to a new EE is maintained well in adulthood in laboratory rodents [27], [30], [33], [38], [43]. For that reason, an alternative interpretation of the current results from the point of view of neural development, i.e. that the results might be possible only in juvenile animals as those used here, is not pertinent. It is important to note that the results obtained after a 2-month EE cannot be explained as a consequence of a loss of novelty in the enrichment protocol, because the environment was completely renewed every 2–3 days, as stated in Methodssection.

The dynamic changes of the cell subpopulations we have analyzed are relevant from the point of view of the role of adult hippocampal neurogenesis. This role is still a matter of strong debate. There is an increasing consensus that immature neurons are directly involved in different kinds of learning and memory, as well as in some types of emotional behavior. However, and despite the tremendous efforts made in the last decades to know the role of the adult hippocampal neurogenesis, not all evidences point to the same direction. Two main streams of evidences can be drawn, but both have long seemed to be apparently contradictory. Numerous reports have studied cognitive performance (i.e. spatial learning and memory) and showed that neurogenesis is directly related and required for some but not all hippocampus-dependent tasks, and not required for tasks not involving hippocampus (for a recent review, see for example [53]). Relevantly, the dependence of some spatial learning paradigms on adult hippocampal neurogenesis has been revealed in two mouse studies using genetic ablation of neurogenesis but not in others [53]. In the same way, the dependence of hippocampus-dependent contextual fear conditioning on neurogenesis has been revealed in two mouse genetic models and by using X-ray irradiation but not in a number of reports by using other methods [53].

These inconsistencies can be due to differences in the mouse strains used, the distinct methods used to increase or decrease neurogenesis, the specific behavioural test design, and not the less important, the time during which neurogenesis rate has been modified, as long as one of the most extended hypothesis in the field states that the different subpopulations of new immature neurons might play different roles as a function of its maturational status. Therefore, the manipulation of distinct subpopulations of immature neurons in different works could lead to completely different results.

This scenario is complicated when some evidences are considered, pointing to a more complex relationship than that previously thought between the immature neurons and some behavioural paradigms. In this line of evidences, some reports have shown that some subpopulations of new neurons must survive while others must die to permit the animals display a normal performance in some behavioural paradigms [54], as well as some works have demonstrated that adult-born neurons appear to facilitate the process of generation of new memories [55], a process probably related to the fact that adult hippocampal neurogenesis modulates the removal/maintenance of long-term potentiation in vivo [56].

Prior to performing the experiments, we injected CldU and IdU at equimolar doses to BrdU into normal mice of the same strain, sex and age as those used here. We checked that with increasing time intervals between the injection of CldU and of IdU in the same animals, fewer double labeled cells were detected, as expected. We also checked that the total number of CldU^+^ or IdU^+^ cells is similar irrespective of whether the first thymidine analogue injected is CldU or IdU, both after 24 h and 2 week survival times (see Methodssection).

We found that an increase in cell survival, through reduced cell death (fewer fractin^+^ cells) could account for the higher cell numbers after long-term EE, even though an increase in proliferation (pH 3^+^ cells) also contributed to this increase at the beginning of EE in naïve mice. It was reported that cell proliferation is increased after 1-day of EE [27]. Accordingly, the numbers of the immature neurons, (DCX^+^/Calret^−−−^, DCX^+^/Calret^+^), increased after one week of EE, as well as after 2 months of EE, as expected. A previous work has also reported an increase of DCX^+^ cell numbers after a 2-month EE while the number of a small subpopulation of these immature cells (12-day old BrdU^+^ neurons) remained unchanged [17]. We found here a similar result considering the 11-to-14 day old IdU^+^ cells in Experiment 2. This is explained by the long time period during which an immature dentate cell can express doublecortin. Therefore, the DCX^+^ cell population is made up of subpopulations of immature cells with a wide range of ages. Thymidine analogs label only a discrete portion of these subpopulations. Alternatively, an enlargement of the period during which an immature neuron in the GCL is expressing doublecortin might account for this result. Further investigation will be needed to dilucidate this issue.

The neuronal markers used in the present work to label the cell subpopulations let us to check the phenotype of these cells. The cell fate of newborn cells in the adult hippocampus has been analyzed in a number of reports (see for example [57]; for a review, see [58]). These reports have shown that the great majority of new cells become granule neurons, while a small proportion is glia. We have analyzed the cell identity of the subpopulation of 4–7 days old cells (IdU^+^ cells) in every experiment (Figure S1). We found that around 80% new cells are doublecortin^+^, i.e., they are immature neurons as expected (similar to that of the majority of works in the field). These percentages were not significantly changed as a result of enrichment. The cell fate of the rest 20% new cells is unknown, and composed by non-differentiating cells and/or glial cells (including perhaps a proportion of cells fully differentiated, as the total time required for newborn neurons to fully differentiate is extremely variable –[59]-).

We consider that whether enrichment has any effect on newborn cell fate, it might play such effect before cells become committed to neurons, and after one week (IdU^+^ cells result, above mentioned) 80% of newborn cells are already neurons. Enrichment did not modify such proportions. Besides, the new cells surviving more than 1 month after birth in the granule cell layer of adult mice are nearly 90% neurons (reviewed by [58]). This cell fate profile remains the same in 3-month old cells [60], and 11-month old cells [61]. We consider that the putative increase enrichment might cause on the cell fate of the rest 10% new cells along the next 2 months of treatment is a quite tiny proportion. Of course, enriched environment or physical exercise might change the cell fate and/or the differentiation status of a small proportion of new cells in the adult dentate gyrus (largely below the unknown 20% new cells other than neurons mentioned above). Although we consider this aspect very interesting, we think that the main findings of the present work are not substantially modified by this point.

To analyze the sensitivity of the neurogenic subpopulation to environmental richness, we measured cell proliferation and survival after termination of EE. We found that the numbers of both 11-to-14 and 4-to-7 day old neurons were not different between control and EE mice after having returned to normal housing conditions for one week, reflecting the rapid response to environmental change. This finding suggests a tight control of the survival of neurogenic subpopulations, in order to regulate the number of new neurons in the GCL. Likewise, cell death also rapidly returned to the basal state. It is noteworthy that the GCL volume, the total number of mature granule neurons and the total number of DCX^+^ cells (mostly DCX^+^/Calret^+^ cells), were significantly higher in EE mice than in controls after termination of EE. The size of these crowded populations needs time to increase significantly (more than one week of EE if we compare Experiments 1 and 2) or to decrease (more than one-week after returning to normal housing conditions after 2 months of EE in Experiment 3). This data is relevant as it is in contrast to the rapid regulation of cell death/survival we found. Certainly, despite the rapid regulation of some cell populations, only the number of DCX^+^/Calret^−−−^ cells was similar to that of controls after the termination of EE. This is relevant because this subpopulation is more immature and smaller than the other subpopulations that remained larger than controls after the change in the environment. It should be considered that differentiating neurons are not equal from a morphological and functional point of view. According to several studies, the morphologically different cell types (DCX^+^/Calret^−−−^ and DCX^+^/Calret^+^ cells) could be considered as functionally distinct cells (see for example [62]. The possible roles that these distinct subpopulations might fulfill in the adult hippocampus must still be elucidated. Nevertheless, further experiments will be necessary to determine whether longer times of normal housing after termination of EE induce a return to baseline levels of all the cell populations increased.

Finally, we analyzed the cells activity to study the broad range of effects of EE. We found that long-term changes in the environment are required to modify the activity as measured as the number of c-fos^+^ cells. By contrast, the area of GAD^+^ boutons increased after a one week period of EE, although no further changes were found after longer-term EE. However, changes in the ratio of vGlut^+^/GAD^+^ boutons have been reported previously, together with long-term reductions in hippocampal neurogenesis [52]. Therefore, further studies will be necessary to understand how these subtle changes might be related to the effects of EE on hippocampal-dependent behaviors.

The results presented here, in conjunction with previous data, can be used to establish a timeline of the effects of EE, with a rapid increase in proliferation and a rapid decrease in cell death from the very first stages of EE, followed by a long-lasting decrease in cell death after long-term EE. However, the rapid increase in short-term cell survival takes effect only in naïve animals that undergo EE, as shown here. When EE is considered in the long-term, the initial increase (24-to-72 hour old cells) and the long-term (≥3 week old cells) increase in cell survival account for the increased neurogenesis and total number of GCL granule neurons. Conversely, the short-term survival of GCL newborn cells is not affected in EE mice compared with controls, except at the very beginning of the enrichment, as demonstrated here. We speculate that the cell environment and the density of the dentate granule neuron population where the new neurons are born act in unknown ways to regulate the survival of the newborn cells. In a different paradigm (treadmill running), it was reported that AHN is modified only in trained mice as a function of the number of existing granule neurons in the GCL [48]. In the present paradigm, as the number of older new neurons increases, that of the younger cells decreases. The numbers of existing new cells in the GCL might act as an endogenous auto-limiting factor for the newborn cells. We propose that this particular regulation of the number of newborn neurons by EE might provoke an increase in cell survival only when EE takes place during the whole immature stage of the new neurons, whereas at the beginning of EE there is an increase in the survival of cells of any age. In this way, environmental richness might have a rapid control on cell number in the GCL, as the termination of enrichment leads to a rapid control of cell death and immature neuron survival (for example, by means of the influence the numerous older neurons may exert on young newborn cells).

## Supporting Information

Figure S1 Cell identity of the subpopulation of 4–7 days old cells (IdU+ cells) in every experiment
(0.06 MB TIF)
