# Supplementary figures and images for: Differential Regulation of the Variations Induced by Environmental Richness in Adult Neurogenesis as a Function of Time: A Dual Birthdating Analysis
Source: PLoS One. 2010 Aug 16;5(8):e12188. doi: 10.1371/journal.pone.0012188 (PMC2922333; doi:10.1371/journal.pone.0012188)

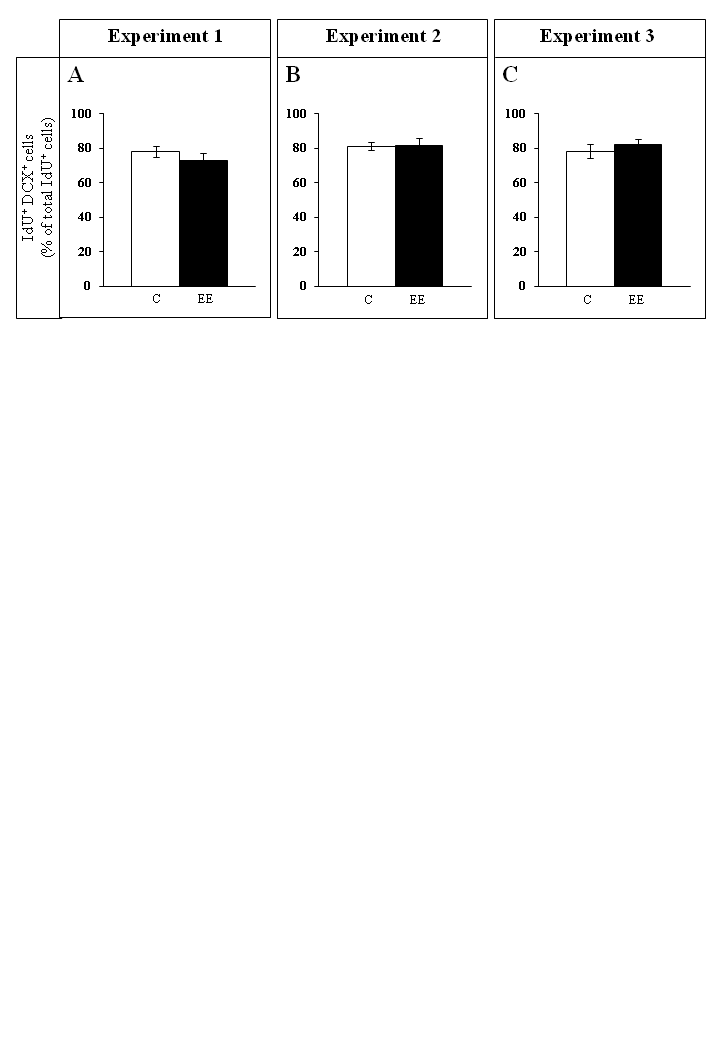

Supplement: Figure S1 — Cell identity of the subpopulation of 4–7 days old cells (IdU+ cells) in every experiment (0.06 MB TIF) [file pone.0012188.s001.tif]
